# Everyday Practices and Activities to Improve Pre-school Self-Regulation: Cluster RCT Evaluation of the PRSIST Program

**DOI:** 10.3389/fpsyg.2020.00137

**Published:** 2020-02-05

**Authors:** Steven J. Howard, Elena Vasseleu, Marijka Batterham, Cathrine Neilsen-Hewett

**Affiliations:** ^1^School of Education, University of Wollongong, Wollongong, NSW, Australia; ^2^School of Psychology, University of Wollongong, Wollongong, NSW, Australia; ^3^Early Start, University of Wollongong, Wollongong, NSW, Australia; ^4^School of Mathematics and Applied Statistics, University of Wollongong, Wollongong, NSW, Australia

**Keywords:** self-regulation, executive function, school readiness, preschool, intervention, RCT

## Abstract

The Preschool Situational Self-Regulation Toolkit (PRSIST) Program was developed as a low-cost and embedded approach for educators to foster pre-schoolers’ self-regulation and related abilities (e.g., executive function, school readiness). This study reports on a cluster RCT study with 50 Australian pre-school services to evaluate the effectiveness of the PRSIST Program for improving children’s self-regulation, executive function and school readiness, compared to current routine practice. Pre-school centers were recruited to reflect the breadth of geography, pedagogical quality, and socio-economic catchment areas across the early childhood education and care sector. All children identified as in their final year of pre-school education at these centers were invited to participate, resulting in a sample of 473 3-5-year-old children at baseline. Centers were randomly assigned to groups after baseline data collection, and data collectors were blinded to group assignment throughout the study. It was hypothesized that engagement in the PRSIST Program would improve children’s self-regulation, executive function and school readiness, over and above normal age-related rates of development. Results indicated small but significant improvements in executive functioning for the intervention group, after adjusting for cluster, baseline results and key covariates. All other outcomes were descriptively in favor of the intervention group but failed to reach significance. Levels of use of the program remained high by most educators throughout the intervention period, suggesting its acceptability and sustainability within these contexts. Together, results show promise for this approach to self-regulation development. Opportunities that might further strengthen this approach are discussed. This study was registered with the Australia and New Zealand Clinical Trials Registry (ACTRN12617001568303) and study protocols published in advance of commencement. Funding for this study was provided by the Australian Research Council’s Discovery Early Career Researcher Award research grant scheme.

## Introduction

For many parents, early childhood educators and professionals, the term self-regulation evokes episodes of exactly the opposite – a child’s dysregulation, which requires their attention and intervention to restore calm to the child and situation (Nixon, 2002; Papadopoulou et al., 2014). Efforts to address these situations are essential, as dysregulations – such as conflict, impulsive behaviors, over-reactions to the situation, and tantrums – are indicative of a child in need, whether temporary or persistent. Unaddressed, these episodes can undermine a child’s relationships with their peers, effective communication with adults and productive engagement in positive developmental experiences (Miller et al., 2010; Williford et al., 2013). Where persistent or severe, dysregulation in childhood increases the risk of developing ongoing behavioral and mental health problems (Althoff et al., 2010; Hyde et al., 2012).

Strategies to foster self-regulation, however, should not begin and end with instances of child dysregulation. For one, efforts to return a dysregulated child to stasis will do little to aid the self-regulation of children not overtly dysregulated and those who “fly under the radar.” For instance, research highlights pervasive gender differences in the manifestation of behavior problems, with girls inordinately channeling this into internalizing problems (e.g., dissociation, surrender, emotional disturbances), while boys more often engage in externalizing behaviors (e.g., hyper-arousal, aggression, acting out; Hodas, 2006). As the latter is likely to be more disruptive and distressing to adults, it is also more often noticed and addressed by educators (Beaman et al., 2007). This may be a contributing factor in the higher rates of specialist referral for boys (Vardill and Calvert, 2000), despite evidence that the prevalence and degree of behavior problems are comparable in girls (McGee et al., 1987; Keenan and Shaw, 1997). Second, reactive and short-lived attempts to address episodes of dysregulation through co-regulation often fail to foster children’s capacity to preemptively – and increasingly independently – control their attention and thinking, behavior, emotional reactions and social interactions in future.

To support and promote genuine *self-*regulation abilities, these capacities should also be fostered in times of good regulation; with children at lower (but often not enough to draw attention), average and higher self-regulation ability. Indeed, any-cause improvements in child self-regulation have been found to be associated with improvements in later-life outcomes even for children initially at average or high levels of self-regulation (Moffitt et al., 2011). This implies that all children could benefit from self-regulation-promoting experiences in the early years. There are few readily accessible programs for educators (or parents), however, to support early self-regulation development beyond instances of dysregulation. Those that do exist often have barriers to access, in terms of cost (requires purchase or expert induction/delivery) or time (supplementing, rather than complementing, the existing daily routines and requirements). The *Preschool Situational Self-Regulation Toolkit (PRSIST) Program* was developed in response – in consultation and collaboration with early years educators and service providers – as a freely and widely available collection of professional learning, supportive adult practices, and child activities that can be embedded within existing pre-school routines. This manuscript reports on a cluster randomized controlled trial (RCT) evaluating the efficacy of the PRSIST Program for the first time.

### The Nature of Early Self-Regulation: Importance and Development

Although there are numerous definitions of self-regulation (Burman et al., 2015), one conceptualization that has come to the fore is self-regulation as the ability to control our attention and thinking, behaviors, emotional reactions, and social interactions, despite any impulses or distractions to the contrary (also termed self-control). In the pre-school years, this includes sustaining attention and resisting distraction, taking turns, persisting with challenging tasks, and initiating or ceasing behaviors that conflict with immediate preference or impulses (e.g., listening to other children in a group activity). The evidence in relation to this formulation of self-regulation is clear: childhood self-regulation abilities robustly predict health, wealth and criminality into adolescence and adulthood (Moffitt et al., 2011). Children with low self-regulation in the pre-school years are more likely to have poorer school readiness and success (McClelland and Cameron, 2011), and poorer health habits and outcomes, socioeconomic position, and mental health in adulthood (Althoff et al., 2010; Moffitt et al., 2011). Research also shows the malleability of early self-regulation, with those children who become more self-controlled achieving better outcomes in later-life (Moffitt et al., 2011). Self-regulation has thus become particularly interesting for researchers, educators and parents, as a means to not only support children’s immediate and pressing needs, but also their long-term outcomes.

There is compelling research supporting the value of ‘earlier’ interventions in forecasting lasting, stable and cost-effective change (Heckman, 2006; Wass et al., 2012). Considered alongside evidence that self-regulation improvements yield benefits not only for children with low self-regulation, but also children at or above age norms (Moffitt et al., 2011), positions scalable early self-regulation interventions as a promising opportunity to improve population trajectories across the lifespan. Despite these compelling findings and possibilities, however, this knowledge has not yet yielded a framework for understanding self-regulatory change, nor has it generated particularly consistent or widespread approaches for enacting this change.

### The Nurture of Early Self-Regulation: Interventions and Programs

Paralleling the diversity in characterizations of self-regulation, there are similarly diverse approaches for attempting to foster children’s self-regulation. One prominent approach to self-regulation intervention derives from a seminal study that reported 4-year-old’s performance on a delay of gratification task – in which the child would receive an enhanced reward if they were able to resist eating a marshmallow for a few minutes – which robustly and longitudinally predicted self-regulation outcomes (Mischel et al., 1989). On the basis of these findings, Mischel et al. (1989) speculated that cognitive and attentional control processes may be essential for successful self-regulation. Decades of investigation that followed has focused on executive functions (EFs) as a core component of self-regulation – via their direction and control of attention, and inhibition of impulses and distractions (Diamond, 2016) – and a means by which to improve diverse outcomes that are associated with good self-regulation. Interventions deriving from this research most prominently features a proliferation of technology-based “brain training” programs, which engage users in activities of increasing cognitive challenge to promote more effective executive functioning. While this approach is now pervasive, programs that adopt this approach can be time- and cost-intensive, and usually necessitate removal of a child for individual sessions that oftentimes require professional administration. The non-routine nature of these programs may constrain their suitability for fostering children’s self-regulation development in their social context. This is evidenced by a typical pattern of findings when adopting this approach: modest gains in EFs and limited transfer of these benefits to untrained tasks, domains and real-world outcomes (Karch et al., 2013; Melby-Lervag and Hulme, 2013).

In contrast to this approach, and expanding on Carver and Scheier’s (1981) feedback loop model of self-regulation, Baumeister and Heatherton (1996) proposed three essential aspects of successful self-regulation: goal selection; sustained motivation to achieve this goal, through reducing discrepancies between current and goal states; and a sufficient capacity to overcome distractions/barriers to achieving this goal. Following from this model, research from education and social psychology has tended to focus on the behavioral, emotional, and social dimensions of self-regulation, such as persistence in challenging tasks, frequency of temper tantrums, and self-directedness (Baumeister and Heatherton, 1996; Hofmann et al., 2012). This has included approaches that foster educators’ self-regulation knowledge and educational supports (Raver et al., 2011), explicit teaching of self-regulation strategies (Flook et al., 2015), embedding activities with self-regulation challenge in children’s daily routines (Tominey and McClelland, 2011) and integrated curricula (Diamond et al., 2007).

These intervention approaches, which target educator practices and classroom curricula, have arguably shown greater promise for improving children’s self-regulation and outcomes (Diamond and Lee, 2011). Indeed, meta-analyses of curriculum-based intervention effects found improved self-regulation after 16 of the 21 curricular programs evaluated and, where available, positive effects on some distal outcomes (Pandey et al., 2018). As one example from this approach, Tools of the Mind (Bodrova and Leong, 2007) is a comprehensive curriculum that embeds EF and self-regulatory challenge within content areas such as literacy and numeracy (Diamond et al., 2007; Barnett et al., 2008). Through its comprehensive programing and schedule, Tools of the Mind directs and supports educators to scaffold children’s higher-order thinking in planning, social learning and play – particularly make-believe play – and, following principles outlined by Vygotsky (1978), gradually withdraw this support with increasing child proficiency. Evaluations of this program have yielded mixed findings (for a review, Baron et al., 2017), although a reconciliation of these results appears to suggest that committed engagement in the Tools of the Mind curriculum can yield positive effects on EF (Diamond et al., 2007) and teacher-reported problem behaviors (Barnett et al., 2008).

Another curriculum-based approach for supporting and enhancing self-regulation is the preschool adaptation of the Promoting Alternative Thinking Strategies (PATHS: Kusche and Greenberg, 1994) program. Rather than a particular and explicit focus on EF skills, as in Tools of the Mind, PATHS focuses on fostering social and emotional knowledge and competencies. Educators engaging with the program are provided with lessons, materials and guidance that focus on topics such as understanding feelings and interpreting social cues. Weekly “circle time” is used to deliver lessons, which are sequenced into thematic units over the year (e.g., compliments, simple versus complex feelings, self-control strategies; Domitrovich et al., 2007). In their evaluation of this program, Domitrovich et al. (2007) found that 3-4-year-old children who were involved in the PATHS program for 9 months had greater emotional literacy, social competency and less social withdrawal than did children in a wait-list control condition. Integration of the PATHS curriculum in the Head Start Research-Based Developmentally Informed (REDI) program in 25 Head Start preschools showed longitudinal benefit: improved academic outcomes for the intervention group in third grade, and improved EF scores for children on low-EF-development trajectories (Sasser et al., 2017).

The Chicago School Readiness Project (CSRP; Raver et al., 2008) takes yet another approach, although is similarly embedded within a comprehensive and structured curriculum. CSRP was designed to enhance school readiness amongst preschool-aged children from low-income backgrounds. To achieve this, CSRP builds early childhood educators’ knowledge of behavior management strategies through extensive professional development and expert coaching from a mental health consultant (Raver et al., 2011). Mental health consultants also provide specialist supports for children with particularly severe self-regulation issues (Raver et al., 2009). While this is a resource-intensive approach to intervention, evaluations of CSRP have reported improved EF, pre-academic skills and teacher-reported behavior problems after less than 12 months of program participation (Raver et al., 2009, 2011).

Curricular approaches seem particularly promising, not only in their ability to generate immediate self-regulation improvements, but also sustained and flow-on impacts after program completion. However, approaches in this tradition are often plagued by time, inflexibility and cost constraints that are prohibitive for many pre-school services and educators (e.g., requires adopting a comprehensive curriculum or intensive program focused on self-regulation). This may be a source of their mixed results, as comprehensive and prescriptive programs may not suit all contexts and educators (thereby impacting program adherence), and effects would not be expected where adherence to program requirements is low (Diamond and Lee, 2011; Wilson and Farran, 2012).

### Theoretical Model of Self-Regulation Change

Another possible explanation for the modest effects of some approaches is their focus on only some elements of self-regulation, which is exacerbated by resource-related barriers to effective, consistent and sustained program implementation (e.g., time, cost, ability to induct new staff). Curricular approaches, for instance, often neglect the role of cognitive control processes for successful self-regulation (although see Tools of the Mind for a curricular approach with this as an explicit focus). Hofmann et al. (2012) propose EFs as the capacity component of self-regulation, providing the cognitive control to direct and sustain attention, remain goal-directed, and override competing interests and distractions. EF “brain training” approaches address this component explicitly, but rarely include components that promote goal setting, motivation and problem solving, which are essential for successful self-regulation according to Baumeister and Heatherton’s (1996) model.

Although this model of self-regulation has yet to be empirically evaluated, it may explain why many existing interventions yield limited transfer to children’s real-world outcomes. That is, while each approach creates conditions necessary for improvement in those abilities (e.g., continual challenge, diversity of intervention activities that are ecologically valid, sustained participation; Diamond and Lee, 2011), they are incomplete in their self-regulatory targets. Given that self-regulatory failure can derive from any one of these aspects (i.e., not selecting a particular self-regulated goal, abandoning progress toward the goal due to weakening motivation or inability to resolve challenges, or insufficient capacity to override contrary impulses and distractions that arise), interventions that support and foster each of these elements may be more likely to succeed. Further, interventions (and their theories of change) must recognize developmental sequences in these abilities, such that capacity and goal-setting components are relatively more constrained in early childhood (and thus require experiences and opportunities that present appropriate but achievable challenge to facilitate development), whereas motivation is often quite high (creating opportunity to leverage this intrinsic interest and motivation toward activities that can promote goal-setting and capacity for control).

From this proposition emerges an approach that acknowledges both the cognitive and socially mediated mechanisms of self-regulatory change. In aiming to foster children’s self-regulation in its social context, this approach should identify those environments, routines and practices that engage and extend (or provide an opportunity to engage and extend) children’s *capacity* to self-regulate. This includes adult practices to promote the conditions for successful self-regulation (e.g., ensure children feel safe and supported, included, and valued), as well as fostering strategies and opportunities for children to *select goals* and experience success in self-regulation (e.g., leading, making choices, planning, experiencing success through effort). To leverage children’s interests and motivation, experiences should be fun and playful. Further, to enhance the likelihood that these experiences yield real-world and everyday improvements, they should be embedded in children’s social contexts. Minimizing the burdens of program induction and implementation – while maximizing program flexibility, educators’ choice and agency, and alignment with current practices and routines – would support implementation and maintenance of the program with minimal additional burdens or resource requirements. Lastly, scalability requires a program that is free, accessible without barriers and can be implemented by those who spend the majority of the time with the children (e.g., parents, educators).

Programs exist that combine at least some of these elements. For instance, the Red Light, Purple Light Circle Time Games Program (Tominey and McClelland, 2011) organizes children into small-to-large playgroups for 20 min, twice per week, during which children play one of five group games that invoke self-regulatory challenge (e.g., doing the opposite of a natural response, such as dancing slow to a fast song). Evaluations have shown feasibility and benefit, such as increases in self-regulation for children initially low in self-regulation (Tominey and McClelland, 2011), and improvement in literacy (Tominey and McClelland, 2011; Schmitt et al., 2015) and math (McClelland et al., 2019). Other programs, such as Kids in Transition to School (KITS), have shown similar success when integrating self-regulation activities (as well as early literacy and prosocial skills) into group activities for children with developmental disabilities (Pears et al., 2015).

Yet the absence of some of the aforementioned criteria may constrain consistency or size of program effects, and/or possibility for widespread program uptake. For instance, given their different theories of self-regulatory change, some theorized components of self-regulation (i.e., goal setting, motivation) are not explicitly targeted through educator practice or child activities. The constrained number and context of self-regulation-promoting situations further limits the everyday situations (e.g., in dyads, in full-group activities, in physically active play) in which children are given an opportunity to practice and extend these abilities. In terms of accessibility, this approach often requires delivery or face-to-face training by a master interventionist, and/or ongoing coaching, which present barriers to access and implementation. Given the successes of this approach and opportunity to empower those who have amongst the greatest opportunity to influence children’s early trajectories, the PRSIST Program was designed to adopt a similar approach but also address these additional criteria.

### Current Study

To address limitations in current pre-school self-regulation intervention approaches, the PRSIST Program was designed with this theory of change in mind. Specifically, the PRSIST Program provides educators with: online professional development, to foster practices that set conditions for optimal self-regulation (i.e., reducing factors that have been shown to undermine children’s self-regulation, such as stress and loneliness; Diamond, 2013), and support children’s goal-setting through choice and success (fostering the goal-setting and motivation elements of self-regulation); and playful small- and large-group activities with embedded self-regulation challenge, to extend children’s capacity to control their attention, behaviors, emotions and interactions (to develop the capacity component of self-regulation). The PRSIST Program was designed in consultation with early childhood educators to ensure its acceptability (to children and educators), flexibility and compatibility with current routines, to maximize the likelihood of sustained program implementation. The PRSIST Program was thus developed as a comprehensive – but flexible, embedded, and readily scalable – approach to support early self-regulation in pre-school contexts. In this initial evaluation study, the PRSIST program was implemented and evaluated with 50 pre-school services, using a cluster RCT design, to determine its effectiveness for improving children’s self-regulation, EF and school readiness outcomes. We hypothesized that children in the intervention group would show greater improvements in self-regulation, executive function and school readiness compared with children in the control (typical pre-school practice) group. To maximize the quality of evidence generated, conduct and reporting of this study follows the CONSORT statement for cluster RCTs.

## Materials and Methods

### Design and Participants

This study was a 6-month, 2-arm cluster randomized controlled trial comparing a pre-school self-regulation program (*PRSIST Program*) with typical practice (control group). Fifty pre-school centers in metropolitan and regional areas of Australia were recruited to be broadly representative of population proportions for geography (84% metropolitan), socio-economic decile for their catchment area (*M* = 5.91, *SD* = 2.24, range = 1–10), and statutory quality assessment rating (i.e., 44% Exceeding, 48% Meeting, 4% Working Toward, 4% unrated against the National Quality Standard). Australia’s early childhood education and care (ECEC) sector includes a range of pre-school provision (e.g., preschool for 4–5-year old children in the year before formal schooling, long-day care services from infant to age 5, family day care) that is delivered by not-for-profit, for-profit or state providers. While there is no state or national curriculum for the Australian ECEC sector, all pre-school services are required to follow the Australian *Early Years Learning Framework*, which outlines expected outcomes of children from birth to age 5. For this study, participating pre-schools: were structurally equivalent in terms of being long-day care services providing care to children aged 2–5 years, up to 5 days/week; were run by community or not-for-profit providers; and had at least one Bachelor-qualified educator (or government waiver).

The focus of the study was the final year prior to formal schooling, which yielded a total of 52 classrooms (most centers had one pre-K room, except for two services that had two). One-hundred and sixty-one educators participated in the study. Characteristics of these educators were broadly consistent with those in the sector: a majority were female (98.8%) and full-time (59.0%); had an average of 10.48 years of experience in the industry (range = 0–36 years) and 4.29 years at their center (range = 0–20 years); and were diverse in qualifications (58 degree, 55 diploma, 41 certificate and 4 no formal qualification).

**FIGURE 1:**
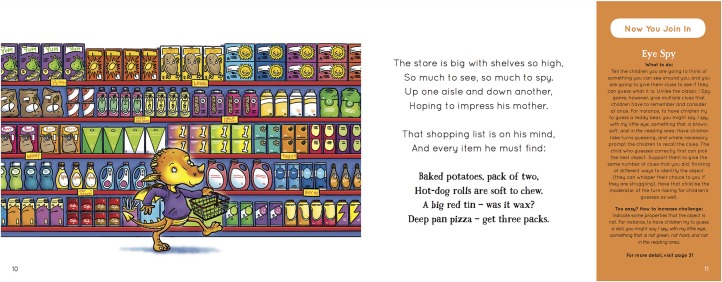
Example story page and linked child activity pertaining to cognitive self-regulation. The picture books were developed so that educators could read the rhyming story to children, and after the story facilitate an activity linked to primary plot points – as a soft entry into doing the activities with children. This example is from a book with a cognitive self-regulation storyline – the main character must remember a shopping list despite distraction and competing interests. The rhyming story and associated image take up the majority of the page, while an activity that can be completed after the story – which is linked to the plot of the current page – is provided in the panel on the right. This image is reproduced with permission of the publisher, Ceratopia Books Ltd.

All children in their final prior-to-school year in these centers, who attended at least one of the 1–2 assessment days, were invited to participate in this study. There were no further exclusion criteria. Parental consent to participate was provided for 547 3-5-year old children, all of whom were identified as likely to be attending school in the subsequent year. The flow of participants throughout the study is depicted in Figure 1. At baseline, 473 of these children were assessed (86.5%), with non-participation largely due to absence on the day of assessment. The mean age of this sample was 4.44 years (*SD* = 0.38, range = 3.20–5.33), with a relative balance of boys and girls (48.2% girls). Children who were identified as of Aboriginal or Torres Strait Islander descent comprised 7.2% of the sample, which is in line with population estimates for this age group (Australian Institute of Health and Welfare (AIHW), 2012). Family income was diverse: 11.9% of families qualified for full childcare benefit subsidies (low income); 65.5% of families qualified for some childcare benefit (low-middle to middle-high income); and 22.7% of families did not qualify for any childcare benefit subsidy (high income). Maternal education levels were also diverse: 9.5% did not complete high school; 9.3% completed only high school; 30.6% had completed a diploma, trade, certificate; 34.6% completed a tertiary degree; and 16.0% a post-graduate qualification. At follow-up, 426 children were assessed, which corresponded to a 90.1% retention rate. Non-participation at follow-up was due to the child having left the center or absence on the day of assessment.

Centers were randomized after baseline data collection, using a computerized random number generator. As such, those involved in recruitment of centers and assessment of children at baseline were unaware, at time of recruitment, to which group centers would be allocated. The trial was registered with ACTRN (ACTRN12617001568303) and protocols were published prior to the trial’s commencement (Howard et al., 2018). The study was approved by the university’s Human Research Ethics Committee, and participants were those whose parents provided informed written consent and themselves provided verbal assent to participate.

### Intervention (*PRSIST Program*)

The *Preschool Situational Self-Regulation Toolkit (PRSIST) Program* aims to engage, challenge and extend young children’s self-regulation in ways that are playful, low-cost, routine, and target each of the aspects required for successful self-regulation (i.e., goal setting, motivation, problem solving, self-regulatory capacity). The PRSIST Program is a collection of professional learning, adult practices, play-based child activities, and home-based resources to support the development of children’s self-regulation. The PRSIST program was designed to be compatible across a range of early learning contexts, but in this study was implemented by pre-school educators. Educators were inducted into the program through hard copy program materials, a program website^1^, and monthly 1-h teleconference calls to highlight different aspects of the program and discuss educators’ experiences and challenges. All program materials are freely available on the program website for inspection, replication, revision or adoption of program elements.

In previous phases of this research, all program elements were piloted, evaluated and revised on the basis of feedback from early years educators (e.g., child and educator enjoyment, program compatibility with pre-school contexts, routines and practices, perceived benefit). In line with this feedback, the program was developed so that it can be flexibly implemented for varying durations, intensities, and using different combinations and sequences of elements.

For the current trial, however, educators were asked to implement the program over the course of 6 months, implementing each of the program’s four core elements described below. While all program elements were made available on completion of baseline assessments, the program elements were explicitly introduced and emphasized in a staged manner, to ensure sufficient foundations for implementation. Specifically, the first month focused on completion of online professional development, the second month on child activities, third month focused on formative assessment and fourth month focused on increasing challenge in child activities. Minimum expectations of engagement in the program were communicated to educators, which are outlined in relation to each program element below.

#### Professional Development (Adult Practices)

Educators were asked to engage with the program’s nine accredited online professional development videos within the first 2 months of the program. These videos, which were drawn from the self-regulation components of the evidence-based Fostering Effective Early Learning (FEEL) professional development (Siraj et al., 2018), introduce the nature and development of early self-regulation, and supportive adult practices. These videos were complemented by a practice manual that describes 11 principles, and associated practices, to support children’s self-regulation development and minimize factors that undermine self-regulation (e.g., stress, sadness). In the manual the principles are described (e.g., foster intrinsic motivation through encouragement), contextualized in a real-life scenario to illustrate its importance (e.g., a child shows an educator a construction they have worked hard on), and specific practices are provided related to the principle (e.g., open-ended questioning).

#### Child Activities

**FIGURE 2:**
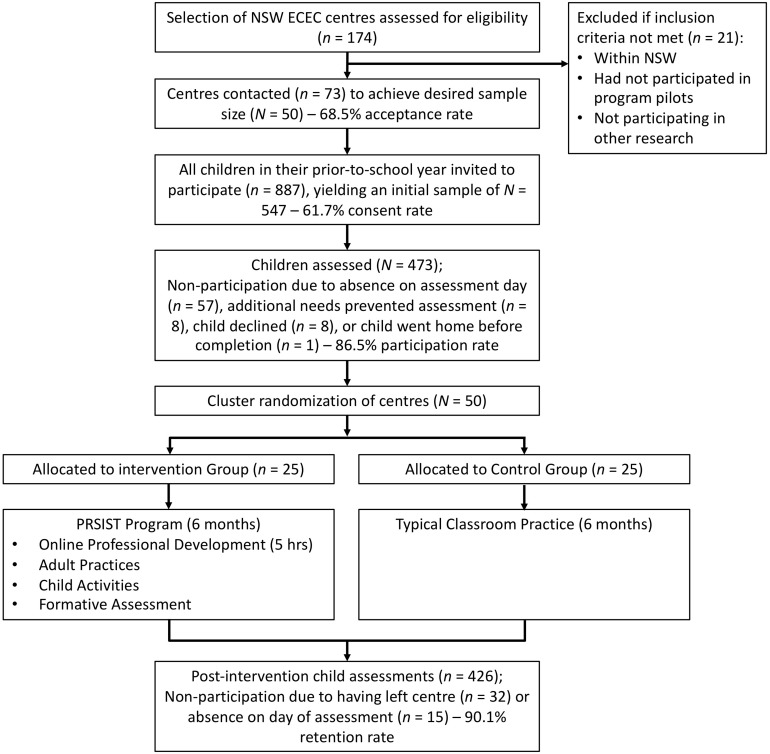
CONSORT flowchart of stages and participants in the study.

In addition to the adult practices, a collection of 28 play-based activities were provided to extend children’s self-regulatory capacity. These activities were developed from: practices already occurring in high quality pre-school services; minimal modification of existing practices (i.e., modified to maximize self-regulatory benefit) in high-quality centers, which were identified as high quality in the FEEL study (Neilsen-Hewett et al., 2019); or newly created activities that were piloted and revised based on the feedback of educators across a range of pre-school services. In addition to being made available online and in hard copy manuals, activities were compiled into a series of children’s books as an easy entry for educators to read about and conduct the activities. The storyline for each book relates to a domain of self-regulation (i.e., behavioral, cognitive, social-emotional), with self-regulation activities linked to central plot points and a full compendium of activities in an appendix at the end of the book (see example at Figure 2).

All activities included instructions for implementation, how to increase the challenge of the activity as children became more proficient, how the abilities required for the activity relate to children’s everyday self-regulation, and links to Australia’s national Early Years Learning Framework. *Disciplined Dance* is one such activity done routinely in early childhood contexts, in which children dance whenever the music plays and stop when the music stops. A common tendency in this game, however, is to either eliminate a child who does not “freeze” (thereby giving the least amount of practice to children who perhaps could benefit most) or ignore that the child continued dancing. In our variation of this activity, all children continue to play the game throughout, but children who do not freeze are aided by removing some of the body parts from consideration. For instance, if a child does not freeze at the first stoppage of music, they kneel for the next round (removing their legs from consideration). If they are unable to freeze again, they sit. Upon successful freezing, the child returns to an earlier position. To make this activity even more challenging, educators reverse the sequence – children must be still when the music is playing, and dance when the music is turned off.

The program was designed so that the timing, intensity, selection, and sequence of child activities is flexible; however, within the current trial educators were asked to complete a minimum of three activities of their choosing per week. While educators were free to select the activities that they and the children enjoyed best, they were encouraged to complete activities of various types and categories each week. Fidelity of this intensity requirement was evaluated through monthly wall-calendar sticker charts, returned to the research team, that showed the date and frequency of each activity.

#### Formative Self-Regulation Assessment

To appropriately plan for and support children’s self-regulation development, information about their current developmental progress in this area is essential. To support this understanding, participating educators were given access to online training for the PRSIST formative assessment tool. This tool involves observation of children as they perform everyday activities, but structures this observation to: (1) focus on key areas of self-regulation; and (2) provide actionable data based on a child’s current developmental progress. Use of this tool was optional, but if used it was recommended that each child be assessed at least twice during the intervention period to support tailoring the complexity of the child activities to children’s current developmental needs.

#### Parent Newsletters

While the trial did not explicitly focus on parental involvement, intervention centers were provided with monthly parent newsletters designed to support the sharing of information and practices with the home. Each of the six newsletters, which were one double-sided page in length, presented information relating to what self-regulation is, its importance in the early years, observing their child’s self-regulation and ideas for supporting self-regulation development in the home.

#### Teleconferences

Directors and educators from intervention centers were invited to join 1-h teleconferences run monthly, at the end of the first, second, third, and fourth months of the program. Participants were invited to participate in any one of three repeat sessions in a given week. Educators who were unable to attend these sessions were offered a one-to-one debrief with a member of the research team. The aim of each teleconference was on: highlighting a particular program element to commence or focus on over the following month; facilitating discussion of educators’ current experiences, challenges and needs in relation to the program; and creating an opportunity for educators to share with each other ideas and opportunities that arose from their engagement with the program. This was also an optional component of the program but had a high level of participation (88% attended at least two teleconference calls).

### Control Group (Typical Practice)

The control group continued with their existing program, which included structured and free play time. Given the prevalence of self-regulatory concern amongst ECEC educators, it is likely that some of these activities targeted self-regulation. Further, it is expected that at least some of the educators would have attended professional development during the trial, and some of this might have concerned self-regulation. However, all of this can be considered current routine practice and represents an appropriately active control condition.

### Measures

Outcomes were measured at the child level and pertained to self-regulation and related abilities (i.e., executive function, school readiness). Given the child activities resembled those routinely enacted in early childhood contexts (e.g., *Disciplined Dance*), and were not designed to approximate the outcome measures, results can be interpreted as near transfer to untrained contexts. The one exception to this was the PRSIST Assessment, which was made available to the educators as part of the program. However, for the purposes of program evaluation this was administered and scored by a trained researcher (rather than educator), and performance indices were not concerned with proficiency in the game *per se* (thereby limiting practice effects).

#### Self-Regulation

The primary outcome was a task that requires complex combination of EFs. *Head-Toes-Knees-Shoulders* (HTKS; McClelland et al., 2014) asks children to remember a correspondence between body parts (e.g., head and knees), and then perform the opposite action to what was indicated (e.g., touch their knees when the facilitator says ‘touch your head’). In doing so it requires children to hold a correspondence in mind (working memory), inhibit the impulse to carry out the action as directed (inhibition), and flexibly switch between correspondences across task levels (cognitive flexibility). The task consists of six practice and 10 test trials at each of three levels: (1) correspondence between head and toes; (2) correspondence between knees-shoulders and head-toes; and (3) flexibly switching between the correspondences of head-knees and shoulders-toes. The task continues until completion or failing to achieve at least four points within a level (such that 2 points are awarded for a correct response and 1 point for a self-corrected correct response). Performance was indexed by the sum of points awarded for all practice and test trials attempted, yielding a score with a possible range from 0 to 94. HTKS has been shown to have good convergent validity with other task- and adult-report measures of self-regulation, predictive validity of academic learning (Ponitz et al., 2009), and psychometric reliability (e.g., α ranging from 0.92 to 0.94; McClelland et al., 2014). Reliability in the current study was similarly strong (Time 1 α = 0.97, Time 2 α = 0.97). Fieldworkers completed the online training module prior to in-field data collection to ensure accuracy of scoring and inter-rater reliability. All other outcomes were considered to be secondary.

*Preschool Situational Self-Regulation Toolkit (PRSIST) Assessment* (Howard et al., 2019) is an observational measure of early self-regulation that engages children in self-regulatory activities, and rates the child’s behavior in relation to cognitive and behavioral self-regulation. The first PRSIST Assessment activity is a memory card game. In this activity children, in a group of four, take turns trying to find a matching pair of cards (e.g., 8 pairs for 4-year-olds, 14 pairs for 5-year-olds), taking around 10 min to complete. The second activity is an individual curiosity boxes activity, in which children are presented with a series of three boxes of increasing size and asked to guess their contents. The sequence of guessing occurs as follows: first, guess based only on the size of the box (no touching); second, guess after gently lifting the box to feel its weight (no shaking); third, guess after shaking the box (no opening); and lastly, guess after closing your eyes and feeling the object inside (no peeking). This activity takes approximately 5 min to complete. Each child’s self-regulation was rated at the end of each activity. Items were scored along a 7-point Likert scale, with the ratings representing a judgment of the frequency and/or severity of behaviors pertaining to cognitive self-regulation (e.g., Did the child sustain attention, and resist distraction, during the instructions and activity?) and behavioral self-regulation (e.g., Did the child control their behaviors and stay within the rules of the activity?). This yielded two sets of ratings per child, which were averaged for the two activities before aggregating into cognitive (six items) and behavioral self-regulation indices (three items) with a possible range from 1 to 7. A full description of this measure and administration protocols are described elsewhere (Howard et al., 2019). To ensure inter-rater reliability, observers completed the online training module – at the end of which an observer rating ensures sufficient inter-rater reliability – and five joint observations of video data alongside a member of the research team prior to in-field data collection. This measure has shown good construct validity, reliability (α ranging from 0.86 to 0.95), and concurrent validity with task-based self-regulation (*r*s ranging from 0.50 to 0.63) and school readiness measures (*r*s between 0.66 and 0.75) (Howard et al., 2019). Reliability in the current study was similarly strong (Time 1 α = 0.92, Time 2 α = 0.90).

Educator-reports of children’s self-regulation on the *Child Self-Regulation & Behaviour Questionnaire* (CSBQ; Howard and Melhuish, 2017) were also collected. This scale consists of 34 items pertaining to the typicality of children’s everyday behaviors (e.g., “Persists with difficult tasks”). Each item was rated by the child’s educator along a 5-point Likert scale from “Not true” to “Certainly true” about the child. Ratings on individual items were averaged to generate subscales of cognitive (five items), behavioral (six items), and emotional self-regulation (six items), as well as subscales concerning prosociality, sociability, internalizing problems, and externalizing problems. The subscales have shown good reliability (α ranging from 0.74 to 0.89) and convergent validity with other adult-report measures of children’s behaviors (Howard and Melhuish, 2017). Reliability in the current study was similarly strong (Time 1: cognitive α = 0.87, behavioral α = 0.88, emotional α = 0.79; Time 2: cognitive α = 0.89, behavioral α = 0.87, emotional α = 0.85). To reduce the number of analyses performed, a single self-regulation index was generated by averaging the three self-regulation subscales.

#### Executive Functions

Individual EFs were indexed by measures of working memory, inhibition, and cognitive flexibility selected from the iPad-based *Early Years Toolbox (EYT*; Howard and Melhuish, 2017). Specifically, working memory was indexed by the *Mr. Ant* task, which asks children to remember the spatial locations of “stickers” placed on a cartoon ant, and identify these locations after a brief retention interval. Test trials increase in complexity as the task progresses (progressing from one to eight stickers), with three trials at each level, until the earlier of completion or failure on three trials at the same level of difficulty. Working memory was indexed by a point score that estimates working memory capacity, calculated as: one point for each level, from the first, in which at least two of three trials are performed correctly; and then one-third of a point for each correct trial thereafter (yielding a possible range from 0 to 8; Howard and Melhuish, 2017). Inhibition was assessed by the *go/no-go* task, which requires participants to respond to “go” trials (“catch fish”) and withhold responding on the “no-go” trials (“avoid sharks”). The majority of stimuli are “go” trials (80% fish), thereby generating a pre-potent tendency to respond that children must inhibit on “no-go” trials (20% sharks). After instruction and practice, 75 test stimuli were presented across three 1-min blocks (separated by a short break and reiteration of instructions). Each trial involved presentation of an animated stimulus (i.e., fish or shark) for 1500 ms, each separated by a 1000 ms inter-stimulus interval. In line with protocols of Howard and Melhuish (2017), inhibition was indexed by an impulse control score, which is the product of proportional “go” (to account for the strength of the pre-potent response generated) and “no-go” accuracy (to index a participant’s ability to overcome this pre-potent response), to yield a proportional accuracy score that ranged from 0.00 to 1.00. Finally, cognitive flexibility was assessed by the *Card Sort* task, which asks children to sort cards (i.e., red rabbits, blue boats) first by one sorting dimension (e.g., color), then switch to the other sorting dimension (e.g., shape). The task begins with a demonstration and two practice trials, after which children begin sorting by one dimension for six trials. In the subsequent post-switch phase, children are asked to switch to the other sorting dimension. For all test items, each trial begins by reiterating the relevant sorting rule and then presenting a stimulus for sorting. If the participant correctly sorts at least five of the six pre- and post-switch stimuli, they then proceed to a border phase of the task. In this phase, children are required to sort by color if the card has a black border or sort by shape if the card has no black border. Cognitive flexibility was indexed by the number of correct sorts after the pre-switch phase (yielding a score that ranged from 0 to 12; Howard and Melhuish, 2017). To more purely index EF (given findings of a single EF factor in the pre-school years, which is impurely indexed by any single task), and constrain the number of planned analyses, an exploratory factor analysis (EFA)-derived factor score was computed for these three EF tasks. Each of these tasks has shown good convergent validity with other task-based measures of EF (*r*s ranging from 0.40 to 0.46) and reliability with children of this age (Howard and Melhuish, 2017). Inter-task correlations in the current sample (*r*s from 0.16 to 0.30) were similar to those previously reported (Howard and Melhuish, 2017), as were correlations with the school readiness measure (*r*s from 0.27 to 0.42).

#### Academic Learning

The academic knowledge of participating children was assessed using the *Bracken School Readiness Assessment (BSRA*, 3rd edition; Bracken, 2007). BSRA is a standardized assessment of areas deemed important for school readiness. It includes subscales of colors (10 items), letters (15 items), numbers/counting (18 items), sizes/comparisons (22 items), and shapes (20 items). For each domain, the assessment continues until completion or three consecutive incorrect responses. BSRA has been shown to be predictive of kindergarten teacher ratings of children’s school readiness and academic results (Bracken, 2007; Panter and Bracken, 2009). Children’s academic learning was indexed by a total raw accuracy score, with a possible range of 0–85.

#### Demographic Covariates

Parents reported on demographic information used as covariates for analyses. These were: child’s age (the date of assessment minus date of birth); child’s sex (1 = male, 2 = female); identification as Aboriginal or Torres Strait Islander; home language (1 = English, 0 = Other than English); a quality of home learning environment (HLE) index from the EPPE Study (Melhuish et al., 2008), which asks about the frequency of eight in- and out-of-home enrichment activities (e.g., reading, sport, extra-curricular activities) to generate a 41-point HLE index; and a postcode-level index of socioeconomic decile created by the Australian Bureau of Statistics (Australian Bureau of Statistics (ABS), 2012), combining census data on factors such as education, household income, and unemployment. This area-level index was used over the family income variable given its increased sensitivity (reported in deciles) over the three wide income bands utilized to capture eligibility for childcare benefit.

### Procedure

All tasks were administered to children in a quiet area of their pre-school center in five sessions across the same day, to maximize children’s attention and minimize fatigue. Measures were administered in the same order to all children, as follows: (1) BSRA; (2) PRSIST curiosity boxes and HTKS; (3) Mr Ant and Go/No-Go; (4) PRSIST memory; and (5) Card Sort. Each session took 10–20 min to complete and were done near the start of children’s final pre-school year (March–April 2018). These assessments were again conducted near the end of the year (October–November 2018), also in a quiet area of the child’s pre-school. All fieldworkers involved in follow-up data collection were kept blind to cluster assignments.

### Data Analysis

To evaluate the effect of the PRSIST Program intervention, data were analyzed using a linear mixed model with a random effect for clustering by center. Unadjusted models and models with sex, age, SES category (low, medium or high SEIFA), HLE index, identification as Aboriginal or Torres Strait Islander (Aboriginal or non-Aboriginal) and language (English or language other than English) are presented. Baseline by group interactions, and interactions between group and sex and group and age were considered for all variables. Data were analyzed using the mixed models procedure in IBM SPSS Statistics (Version 25, IBM Corp., Armonk, NY, United States).

## Results

### Fidelity Checks

Adherence to intervention participation thresholds was evaluated in terms of educators’ completion of the online professional development modules and having engaged children in a minimum of three child activities per week. Engagement with optional program components (i.e., use of formative assessment tool, participation in monthly teleconference calls) was also captured. Educators’ engagement in the online professional development was captured via log in and tracking functionality of the professional development modules. Of the 25 intervention centers, 20 services (80%) had at least one educator complete the professional development within the first 3 months of the intervention period (20% of the services had more than one educator complete the professional development during this time). Type and frequency of child activities each month was captured through a custom-designed activity sticker calendar, which was returned monthly to the research team. On average, six of the program’s self-regulation activities were facilitated with children each week across the intervention period, ranging from none per week to 22 per week. Further, the charts indicated the suggested diversity of activities was met by most centers in most weeks, and certainly over the duration of the program (by centers who engaged with the child activities).

Use of the formative assessment tool was not required, but educators at 16 of the centers completed the online formative assessment training module and successfully completed inter-rater reliability checks. Seven of these centers reported using the tool, while nine reported they had not yet used the tool. Five centers attempted the online training module but did not achieve the required level of inter-rater reliability and had not yet re-attempted the training. Four centers did not attempt the formative assessment training. Attendance at teleconference calls also was not mandatory, yet all except three centers joined at least one of the monthly teleconference calls (eight centers attended two calls, seven centers attended three calls, four centers attended all calls).

Based on these patterns of participation, 20 services (80%) were deemed to have met or exceeded the minimum threshold of participation (i.e., completed the professional development modules and met the minimum of three child activities per week). Those that did not participate in the program were a result of: preparations for government assessment and rating (*n* = 1); substantial illness, maternity leave or turnover of key staff that precluded participation (*n* = 2); or low- or non-participation for undisclosed reasons (*n* = 2). Two of these five centers did not participate in any program elements. The other three centers did not engage with professional development modules or induction teleconference call yet completed child activities. Overall, there were good levels of adherence to the program, especially amongst those centers without significant sector-imposed impediments to participation.

### Intervention Efficacy

The Intra-Class Correlations (ICC) for all outcome measures were small – HTKS ICC = 0.02; PRSIST ICC = 0.08; CSBQ ICC = 0.08; EF ICC = 0.01; Bracken ICC = 0.05 – yet still advocated adjusting for nested data (Hox et al., 2010). Unadjusted and adjusted mean differences between the control and intervention group are shown in Table 1. For both the unadjusted analysis (accounting for clustering and baseline results only) and the adjusted analysis (additionally adjusting for sex, age, SES, HLE, ethnicity, and home language) there was a significant effect of the intervention on executive functioning. This result indicated significantly improved executive function in the treatment group, beyond typical age-related change (indexed by the control group), with an unadjusted mean difference of −0.16; a small yet significant effect, that indicated a negative change in the control group that was significantly greater than the positive effect in the treatment group (Table 1). Baseline by group interactions were conducted to evaluate whether effects differed by baseline levels of self-regulation but were not significant for any of the models (*p*s ranging from 0.101 to 0.834). Interactions between group and gender to determine whether effects differed by child gender (*p*s ranging from 0.121 to 0.937), and group by age to determine whether effects were conditioned by the child’s age (*p*s ranging from 0.123 to 0.770), were not significant for any of the models (Table 1). All outcomes were directionally in favor of the intervention group (indicated by a negative unadjusted and adjusted mean difference) but did not reach significance. A table of correlations between all outcome measures is provided at Table 2.

**TABLE 1:** Unadjusted and adjusted mean differences (95% CI) between control and treatment groups.

	Control baseline *M (SD)*	Control follow-up *M (SD)*	Treatment baseline *M (SD)*	Treatment follow-up *M (SD)*	Unadjusted mean difference (95%CI)	*P* value	Adjusted mean difference (95% CI)	*P* value	Effect size partial eta squared	*P* Value Group × Sex	*P* Value Group × Age
HTKS	23.06 (24.09)	41.83 (27.45)	21.48 (24.07)	42.87 (26.82)	−2.22 (−8.16,3.72)	0.455	−2.10 (−8.85,4.65)	0.533	0.003	0.710(Int)	0.135(Int)
										0.469(Group)	0.118(Group)
PRSIST	7.60 (2.26)	8.46 (2.15)	7.66 (2.01)	8.87 (1.97)	−0.39 (−0.93,0.15)	0.154	−0.46 (−1.05,0.13)	0.138	0.012	0.691(Int)	0.510(Int)
										0.124(Group)	0.668(Group)
SchRd	47.88 (15.55)	58.67 (14.15)	48.08 (16.05)	59.24 (13.57)	−0.39 (−2.25,1.47)	0.676	−0.85 (−2.91,1.21)	0.412	0.006	0.521(Int)	0.770(Int)
										0.297(Group)	0.707(Group)
CSBQ	0.01 (1.04)	−0.09(1.00)	0.03 (0.87)	0.07 (1.00)	−0.18 (−0.42,0.06)	0.139	−0.17 (−0.42,0.08)	0.172	0.005	0.121(Int)	0.123(Int)
										0.053(Group)	0.185(Group)
EF	0.09 (0.791)	−0.06(0.765)	0.03 (0.72)	0.07 (0.70)	−0.16 (−0.30,−0.03)	0.017	−0.16 (−0.31,−0.02)	0.029	0.016	0.937(Int)	0.581(Int)
										0.113(Group)	0.732(Group)

*Means and SD at baseline and follow-up represent mean values not accounting for clustering. The unadjusted mean difference accounts for clustering only, without covariates. Adjusted mean difference accounts for clustering and covariates: sex, age, SES category (low, medium or high SEIFA), HLE index, identification as Aboriginal or Torres Strait Islander (Aboriginal or non-Aboriginal), language (English or language other than English) and baseline values. Partial eta-squared is based on an ANCOVA with all covariates. HTKS, Head-Toes-Knees-Shoulders; PRSIST, Preschool Situational Self-Regulation Toolkit Assessment; SchRd, Bracken school readiness assessment; CSBQ, Child Self-Regulation & Behaviour Questionnaire; EF, latent factor scores for combination of Mr Ant, Go/No-Go, and Card Sort assessments. Analyses included *n* = 217 children in the intervention group and *n* = 209 children in the control group (except CSBQ, for which an additional six questionnaires for children who were absent for direct assessment, and EF, for which five intervention children and four control children did not complete one EF measure).*

**TABLE 2:** Correlations between outcome measures at baseline.

		1	2	3	4	5	6	7	8	9
1	HTKS	–	0.41*	0.31*	0.32*	0.17*	0.35*	0.31*	0.42*	0.52*
2	PRSIST Assessment		–	0.35*	0.41*	0.31*	0.43*	0.37*	0.29*	0.39*
3	CSBQ – Cog. SR			–	0.63*	0.46*	0.30*	0.34*	0.25*	0.40*
4	CSBQ – Behav. SR				–	0.66*	0.28*	0.39*	0.16*	0.28*
5	CSBQ – Emo. SR					–	0.12*	0.20*	0.07	0.12*
6	EYT Mr Ant (WM)						–	0.30*	0.28*	0.38*
7	EYT Go/No-Go (Inhibition)							–	0.16*	0.27*
8	EYT Card Sort (Shifting)								–	0.42*
9	Bracken School Readiness									–

HTKS, Head-Toes-Knees-Shoulders task; CSBQ, Child Self-Regulation & Behaviour Questionnaire; SR, self-regulation; EYT, Early Years Toolbox; WM, working memory. *p < 0.05.

## Discussion

This program of research sought to design, implement and evaluate a program to support young children’s self-regulation development, the product of which was the PRSIST Program. The PRSIST program was developed by reconciling insights from interviews and observations of educators with research-based understandings about the nature, development and change in self-regulation. After pilot and revision of intervention components with educators, the current cluster RCT evaluation of the program over a 6-month intervention period indicated small but significant improvement in EF for the intervention group. All other outcomes (self-regulation, school readiness) also showed descriptively greater improvement for the intervention group, although these changes did not reach significance. In the context of the short intervention period, during which the program was incrementally introduced, implemented and mastered, this pattern of results suggests promise and future enhancements for the PRSIST approach to fostering children’s self-regulation in the pre-school context. Fidelity data further demonstrated that educators were willing and able to implement each of the program’s components over a sustained period of time.

The small but significant positive effect of this intervention on children’s EF – over and above the already rapid development of these abilities in the pre-school years (Anderson and Reidy, 2012) – is consistent with evidence of the ability to support and enhance children’s EFs more broadly (Diamond and Lee, 2011), and preliminary evidence in favor of an embedded practice approach more specifically (e.g., Tominey and McClelland, 2011; Howard et al., 2017). The PRSIST Program contrasts more prevalent EF training approaches, which are often constrained to particular ages (typically older children, adolescents, and adults), contexts (individual, commonly requiring professional administration) and resource availabilities (e.g., time, cost). The current approach represents a low-cost and embedded alternative to these approaches that can be applied as a ‘menu’ of practices, activities and resources to flexibly suit different contexts. That the program’s child activities involve real-world application of cognitive, behavioral and social-emotional control, rather than the targeted training of individual EFs specifically (e.g., through practicing computerized EF tasks; Blakey and Carroll, 2015), minimizes the possibility that improvements are an artifact of task-based learning (Shipstead et al., 2012).

While the primary outcome for the evaluation was child self-regulation, and there was a descriptively greater improvement in self-regulation in the intervention group for all indices, results for this outcome were non-significant. This contrasts other curricular approaches, which have successfully achieved improvements in indices of self-regulation after similar or longer intervention periods (Pandey et al., 2018). There was also a lack of significant improvement in academic knowledge, as an indicator of school readiness. While some studies have shown significant improvement in self-regulation and academic outcomes after intervention (Schmitt et al., 2015; Pandey et al., 2018), others found these outcomes difficult to shift over and above the rapid age-related change already occurring during the pre-school years (e.g., Tominey and McClelland, 2011).

In relation to the PRSIST Program, there are a number of possible explanations for this result. First, given self-regulation develops rapidly in the pre-school years (Montroy et al., 2016), the program may have been insufficient in intensity (e.g., minimum of only three child activities per week, lack of checks that child activities were modified to increase challenge as children improved in competency) and/or breadth (e.g., focus on fostering self-regulation during times of good regulation, with less emphasis on episodes of dysregulation) to outpace this typical developmental trajectory. Indeed, curricular approaches tend to involve more comprehensive and structured programs (e.g., Tools of the Mind), and/or provide more intensive supports (e.g., CSRP), in contrast to the PRSIST approach of providing practices and activities to complement current programing and curricula. While it is possible that the lack of consistent effects was related to insufficient program intensity or breadth, this does not articulate well with significant improvement in EF – cognitive capacities underpinning self-regulation – which also develops rapidly over the course of the pre-school years (Anderson and Reidy, 2012).

Second, it is possible that insufficient quality of implementation generated an estimate of program effectiveness (i.e., when implemented at scale), rather than efficacy (i.e., under the most rigorous and controlled conditions). This was an explicit decision from the outset of this study, given the goal of identifying low-cost, accessible and sustainable approaches that can be employed by pre-school educators. While it might be the case that effects would be more pronounced if the program were implemented with fidelity by members of the research team visiting centers, this would render the approach expensive and difficult to scale. A compromise between these options, however, could involve mentoring and coaching, which may expedite and strengthen educators’ self-efficacy and fidelity in implementation. Indeed, there is ample evidence for the effectiveness of mentoring and coaching when attempting to influence the practices of the current educator workforce (Lambert et al., 2015), and this form of induction is a common feature of other curricular approaches (Barnett et al., 2008; McClelland et al., 2019). However, further research is needed to evaluate whether similar benefits would confer if applied to the PRSIST Program.

Third, it may be the case that children require greater duration and intensity of exposure to the program’s components to detect a self-regulation effect (Diamond and Lee, 2011). In the current study, children in participating centers attended their service an average of 3 days/week (consistent with national enrollment patterns), limiting their opportunities for participation in the program. This was exacerbated by high levels of staff turnover that characterize this sector. Further, the program was incrementally introduced over the 6-month intervention period: i.e., the first month focused on completion of online professional development; the second month on child activities; the third month on formative assessment; and the fourth month on increasing challenge in the child activities. As such, educators’ implementation and mastery of program components was likely incomplete until at least halfway through the 6-month intervention. It may thus be that children who receive longer and more frequent exposure to the program could achieve greater and clinically significant improvements in self-regulation. In the current instantiation, effects were limited to EF and are best characterized as small. Further research is required to evaluate a dose-response effect. There is also potential for latent effects in measured and unmeasured variables, which take time to manifest (c.f., Duncan et al., 2018). Examples may include adjustment and peer relationships upon school entry, and later academic learning following time to exert newly acquired proficiencies and capacities (e.g., EF). Longitudinal follow-up is planned to explore this possibility.

Inability to conclusively and exclusively provide evidence for one of these possibilities, however, highlights limitations within the current study. That is, although the evaluation was rigorously designed and executed according to CONSORT guidelines, funding considerations limited the roll-out and intervention period to only 6 months. It is possible that a full year of program implementation would yield stronger program effects (see, for example, Schachter, 2015). It is also possible that program effects would be strengthened with stricter adherence to high-quality program implementation. While fidelity data indicate good compliance in the frequency and timing of program elements, data are insufficient to evaluate the integrity with which program elements were implemented. While in-person or video fidelity checks were not possible in the current study, this would help monitor adherence. As a researcher-implemented model of delivery would violate our aspiration for a low-cost and barrier-free resource for educators, a plausible middle ground might be a coaching model that supports educators in implementation and adaptation of the program in their context. Lastly, the program was designed with the intention to foster self-regulation in all children, and thus did not focus on instances of dysregulation. However, it is clear that child dysregulation remains a significant concern for educators (Neilsen-Hewett et al., 2019), and future iterations of the program would do well to more explicitly provide support for these children. In guiding such an expansion of the program, there is evidence that children with frequent and severe dysregulation require a different approach to fostering self-regulation, as demonstrated successfully in trauma-informed practice approaches (Holmes et al., 2015). Future studies would also do well to consider implications of differing educator qualifications and experience, whereby different types and levels of support may be needed at varying levels of behavior challenges and educators’ skills to address these.

This study provides preliminary support for some acute benefits of the PRSIST Program, in terms of improving children’s EF, as well as identifies opportunities for further development of the program (e.g., further and differing approaches/supports for children experiencing high frequency or severity of dysregulation; evaluating additional benefits of educator coaching). The specific promise of this approach is further highlighted by its compatibility with pre-school contexts and routines. The flexibility of the program permitted educators to engage with online professional learning at their convenience, implement adult practices aligned to their specific needs, and select and scale child activities that were best enjoyed by children in their center. Acceptability of the program was evidenced by high levels of educator adherence to minimum program requirements – often exceeding these requirements – and, in cases where centers did not engage with the program, this was due to known sector-related issues (statutory rating, staff absence/turnover). The PRSIST Program is not intended to be a complete collection of practices and activities that could support children’s self-regulation, but rather serves as a stimulus from which educators can expand these options. The accessibility and acceptability of the current approach creates a unique opportunity for embedded practices that yield benefits for young children, including those in less-advantaged contexts that are often most in need of support (Diamond and Lee, 2011; Diamond, 2013).

## Data Availability Statement

The dataset for this manuscript is not publicly available because ethics approval was not sought or granted for such use. Requests to access the dataset should be directed to SH at stevenh@uow.edu.au.

## Ethics Statement

The studies involving human participants were reviewed and approved by the University of Wollongong, Human Research Ethics Committee – Social Sciences. Written informed consent to participate in this study was provided by the participants’ legal guardian/next of kin.

## Author Contributions

SH conceptualized the study, secured funding for the study, designed the child activity aspects of the intervention, oversaw data collection, was involved in data analysis, and led writing of the manuscript. EV aided in conceptualizing the study and design of the intervention, managed data collection and entry, and contributed to drafting of the manuscript. MB contributed to evaluation design and planning, led data analysis, and contributed to drafting the manuscript. CN-H led the pedagogical aspects of intervention design and delivery and contributed to drafting of the manuscript.

## Conflict of Interest

The authors declare that the research was conducted in the absence of any commercial or financial relationships that could be construed as a potential conflict of interest.

## Footnotes

1 www.prsist.com.au
